# Asia Oceania Journal of Nuclear Medicine & Biology; A big step forward to advance scientific publication & cooperation in the region

**DOI:** 10.7508/aojnmb.2013.01.001

**Published:** 2013

**Authors:** Seyed Rasoul Zakavi

**Affiliations:** Editor in chief

Asia Oceania Journal of Nuclear Medicine & biology (AOJNMB) is the official publication of Asia Oceania Federation of Nuclear Medicine & Biology (AOFNMB). When publication of an official journal of AOFNMB was suggested in 10^th^ AOFNMB meeting in Tehran and followed to bring into the reality, almost uniformly positive feedbacks from the scientists and colleagues convinced me that it has been an important neglected regional desire. Now it is my great pleasure and honor to serve as editor in chief of this new promising journal.

AOJNMB focuses on issues of interest of scientists and physicians working in the field of nuclear medicine and biology and publishes a variety of articles including original research, technical notes, case reports, review articles and meta-analysis. Although the journal tries to provide a platform for communication between the scientists (in particular from the region) and give them an opportunity to publish their work free of charge, manuscripts from all around the world is most welcome.

AOJNMB is an open access journal with absolutely no costs to authors and their institution. Although open access policy is getting popularity in recent years, the current situation is not in the best interest of authors (1). In some open access journals the publication fee is more than doubled in last few years and increasing by the time. In the era of cost containments and recent economic crisis, the publication fee is imposed in some previously free journals too. This makes free publication more and more difficult for the scientists from developing countries (1). AOJNMB is fully sponsored by Nuclear Medicine Research Center in Mashhad University of Medical Sciences and provides an opportunity for authors to publish with no costs and enjoy the benefits of open access policy simultaneously.

The journal will be published biannually (in May and November each year) for the first two years and may increase the number of issues per year later. The journal uses a standard peer review process and all manuscripts are reviewed by two reviewers for the originality, relevance, quality and integrity of the manuscript. The revised manuscript is sent to a comparative reviewer for further evaluation. Final decision on acceptance or rejection of the article is done by editor in chief based on these expert opinions. Three experienced associate editors and a group of well known editorial board members are serving the journal, which are mainly from Asia Oceania region. I would like to thanks all my colleagues and editors that helped us in this way, especially Prof. Henry Bom and Prof. Seigo Kinuya for their enthusiastic support.

To the extent of my search, 24 journals are published in the field of nuclear medicine and at least 20 are indexed in Scopus or Science citation index. Journal of Nuclear Medicine from North America and European Journal of Nuclear Medicine & Molecular Imaging (EJNMMI) from Europe have the highest impact factors among all nuclear medicine journals(2). Besides these continental journals there are plenty of national journals of nuclear medicine as well. From North America, Seminars in Nuclear Medicine, Nuclear Medicine and biology, Clinical Nuclear Medicine and Journal of nuclear cardiology are well known. From European side, Quarterly Journal of Nuclear Medicine, Nuklearmedizin, Nuclear Medicine Communication, Revista Espanola de Medicina Nuclear, Medecine Nucleare, Nuclear medicine review as well as Hellenic Journal of Nuclear medicine are published for many years. Alasbimn journal of nuclear medicine from South America has been publishing for about 16 years. In Asia Oceania region, Annals of Nuclear Medicine, Korean Journal of Nuclear Medicine, Kaku Igaku, Indian Journal of Nuclear Medicine, ANZ nuclear medicine journal, Molecular Imaging and Radionuclide Therapy and Iranian Journal of Nuclear Medicine are among the oldest journals in this field which have been publishing for more than 20 years. Comparing impact of these journals in nuclear medicine either using Impact Factor or SCImago ranking, revealed that the highest impacts belong to the journals with international or continental coverage (2).

Asia Oceania region as the largest continent with largest population could have a bigger impact on the scientific progress in nuclear medicine. AOJNMB could play an important role in this way and could provide a new opportunity for researchers in developing countries for free publication of their works. It could also potentially make more communication between the scientists of the region with subsequent multicenter research projects and collaborative efforts. Nuclear medicine facilities and resources are not uniform in this region, neither is the scientific publications (3). A quick review of major journals shows that publication from Asia Oceania region is a significant part of global scientific production. Interestingly the growth rate of scientific production in this area is higher than other parts of the world too (4). So we could expect more scientific production from this area in the following years. EJNMMI published 191 original articles in year 2012 mainly from European countries from which 25 articles were from Asia Oceania region (5). In year 2011, 31/190 original articles in EJNMMI were from Asia Oceania region (6). This data should be interpreted in the light of low acceptance rate of articles in EJNMMI that was only 19% in these years. The present data shows a big potential of the Asia Oceania Region in nuclear medicine researches. AOJNMB tries to help nuclear medicine physicians and scientists (in the world in general and in Asia Oceania region in Particular) to publish their works free of charge and make a bigger impact in the scientific communications.

The first issue of the journal enjoys from different variety of articles including 4 original clinical studies, 1 original article in radiopharmacy, one review article and one case report. Also an editorial is written by the president of AOFNMB about history and perspectives of the AOFNMB as well as another editorial by editor in chief.

It is clear that willingness of each member of the Asia Oceania Federation of Nuclear Medicine and Biology is the main requirement for success of this journal. I am confident that AOJNMB will grow to be one of the most important journals in the field of nuclear medicine and biology. Let’s turn it to reality.
